# Preperitoneal vas deferens infiltration in high‐risk prostate cancer

**DOI:** 10.1002/bco2.289

**Published:** 2023-09-17

**Authors:** Ali Serdar Gözen, Antonios Koudonas, Samet Senel, Maurizio Colecchia, Jens Rassweiler

**Affiliations:** ^1^ Department of Urology Medius Kliniken Ruit Ostfildern Germany; ^2^ First Department of Urology, School of Medicine Aristotle University of Thessaloniki Thessaloniki Greece; ^3^ Department of Urology Ankara City Hospital Ankara Turkey; ^4^ Department of Pathology Vita Salute University San Raffaele Milan Italy; ^5^ Department of Urology Danube Private University Krems Austria

**Keywords:** invasion, local extension, preperitoneal space, prostate cancer, vas deferens

## Abstract

**Objectives:**

The objective of this study is to evaluate the prevalence and the importance of preperitoneal vas deferens (VD) infiltration in high‐risk prostate cancer (PCa).

**Patients and Methods:**

In this prospectively designed study, we included 332 high‐risk PCa patients with a Briganti score >5%, who were treated by robot‐assisted radical prostatectomy between July 2017 and February 2022 at the Urology Department, SLK Kliniken Heilbronn. In addition to the standard histological analysis of the distal VD, which was attached to the prostate specimen, we analysed the infiltration status of preperitoneal VD in this cohort. The preperitoneal VD, which represents the middle part of ductus deferens and extends between the internal inguinal ring and obturator fossa, was resected during extended pelvic lymphadenectomy. Distal and preperitoneal VD status was registered together with preoperative and postoperative disease characteristics. Descriptive analysis methods and logistic regression analysis were used.

**Results:**

Briganti score of the target cohort had a median value of 19%, while 235 patients (70.8%) of the group demonstrated a locally advanced disease. The Grade Group at prostatectomy specimen was at least 3 for 286 patients (86.1%). Distal VD infiltration was found in 20 patients (6%) and preperitoneal VD infiltration in two patients (0.6%). Distal VD infiltration was not associated with an increased possibility for positive surgical margins or nodal status among pT3b patients, while both patients with preperitoneal VD infiltration were characterized by highly aggressive disease in locally advanced stage and bilateral distal VD infiltration.

**Conclusions:**

PCa extension along VD may reach a more proximal point of VD than the reported from the existing data infiltration of VD adjacent to seminal vesicles. This rare manifestation of PCa local extension may be the intermediate step to the rare cases of recurrence in the testicles. However, more robust data are needed to confirm the aforementioned hypothesis. Distal VD infiltration seems to have no additional prognostic value among patients with infiltrated seminal vesicles.

## 1 INTRODUCTION

Infiltration of distal vas deferens (VD) adjacent to the seminal vesicle represents a rare form of locally extended prostate cancer (PCa). The prognostic significance of distal VD infiltration (VDI) remains unexplored. A prior study reported a 1.5% occurrence of positive margins at the VD transection site. Notably, 39% of these instances exclusively involved the distal VD level. All VDI cases exhibited features of locally advanced disease, with a predominant co‐occurrence of VDI and ipsilateral seminal vesicle invasion (SVI).^1^ In another study, oncologic surveillance data of a patient cohort with SVI (pT3b) showed that the patients with additional involvement of distal VD had worse oncological outcomes compared with patients with only SVI. In this study, the effect of distal VDI on biochemical recurrence remained significant after adjustment for other negative prognostic factors.^2^ In the more recent literature, there were reports of disease recurrence at the distal VD level, discovered meta‐chronously after primary therapy through novel imaging methods, mainly positron emission tomography/computed tomography (PET/CT) with prostate‐specific membrane antigen (PSMA)‐targeted radiotracers.^3^, ^4^ However, up to the present, there are no recommendations about the histological analysis of VD, and their sampling is not considered obligatory.^5^

Moreover, the involvement of more proximal parts of VD in the pathological result of radical prostatectomy has not been reported until now. This pattern of extension may be developed through the lymphatics or the lumen of the VD, which represents an additional way of PCa local extension and metastasis. Proximal VDI could be associated with PCa recurrence events, the unusual manifestation of testicular metastases in PCa patients, and the shortening of disease‐free survival and cancer‐specific survival after definitive therapy. In the context of therapeutic and prognostic consequences of this rare form of PCa local extension, we investigated the prevalence of proximal VDI combined with the distal VD status in the prospectively collected data of our department.

## 2 PATIENTS AND METHODS

### 2.1 Patients

**FIGURE 1:**
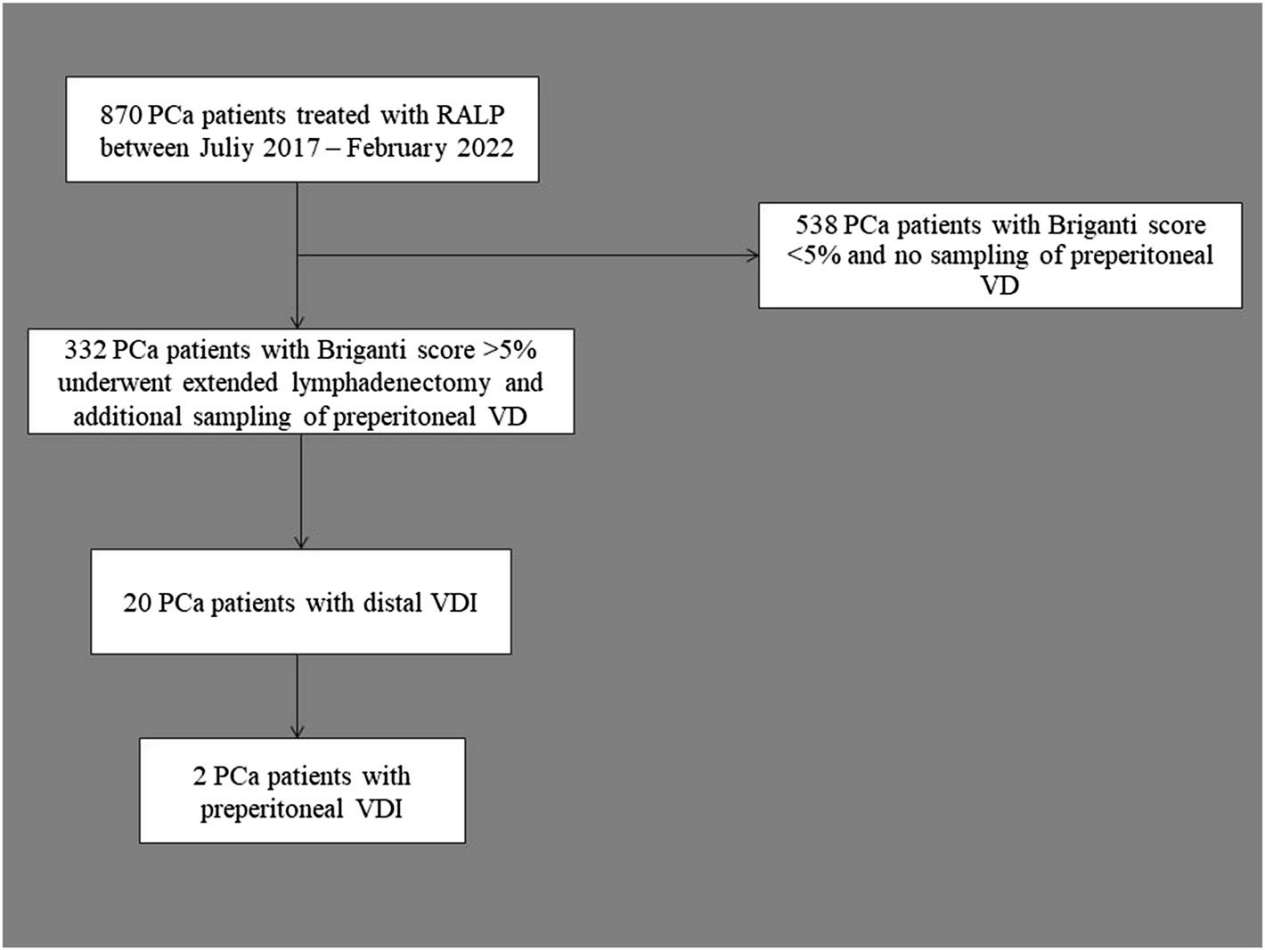
Flow chart illustrating the patient inclusion in the study. PCa, prostate cancer; RALP, robot‐assisted radical prostatectomy; VD, vas deferens; VDI, VD infiltration.

**FIGURE 2:**
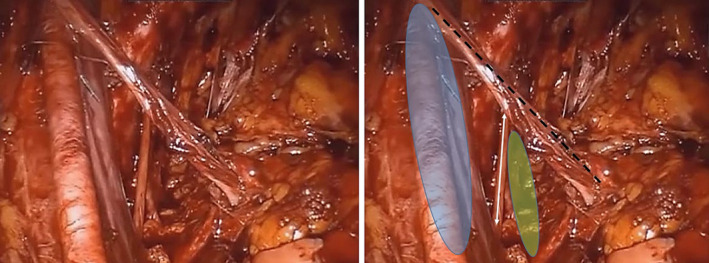
Intraoperative view of the left preperitoneal vas deferens (black dotted line) and adjacent anatomical structures (blue coloured area: left external iliac vessels; green coloured area: fossa obturatoria; and white double arrow line: obturator nerve).

Between July 2017 and February 2022, 870 consecutive patients with nonmetastatic PCa underwent robotic‐assisted radical prostatectomy (RALP) in our department. Among them, in a subgroup of 332 patients with increased risk for lymphogenic extension, as calculated with a Briganti score >5%,^6^ we performed extended pelvic lymphadenectomy (ePLND) (Figure 1). During this operative step, we resected and collected the middle part of VD, which is normally divided as a standard manoeuvre during ePLND.^7^ This part of VD is defined as preperitoneal VD and extends between the internal inguinal ring and fossa obturatoria at both sides, in the anatomic space known as the space of Bogros (Figure 2). Additionally, every pair of distal VD was sent for histological examination as part of the prostate specimen. All operations were performed by two surgeons in our department (J.R. and A.G.).

Tissue sampling and data collection were performed prospectively and evaluated retrospectively after approval of the study protocol from the ethics committee (E2‐22‐2683). Clinical data included age, prostate‐specific antigen (PSA) at the time of diagnosis, clinical staging, pathological staging, the Grade Group at biopsy and final pathology, surgical margin status, prostate volume at the transrectal ultrasound, and surgical specimen, tumour volume, nodal involvement status, and distal and preperitoneal VD status. Briganti score was calculated according to the 2012 model.^6^ The staging was performed according to the AJCC8^th^ edition TNM staging system.^8^

### 2.2 Methods

All tissue specimens were analysed by two experienced pathologists in our institution, who were blinded to the clinical information of the patients. Descriptive statistics were applied for the characterization of the high‐risk patient group with available preperitoneal VD pathology, and the median values were reported for the continuous variables because these variables were not normally distributed. Logistic regression was applied for examining the association of distal VD positivity with other adverse histological findings (nodal disease or positive surgical margin). All statistical analyses were performed using SPSS 25.0 software (IBM SPSS Statistics, IBM Corporation, Chicago, IL).

## 3 RESULTS

The patient group, which underwent separate histological examination for preperitoneal VDI, included 332 patients with a median age of 68 years. The median PSA concentration at diagnosis was 10.3 ng/mL and the Briganti score had a median value of 19%, demonstrating a relatively high possibility of lymph node involvement. High‐risk preoperative features were verified from the pathology report, as the majority of the tumours was extended beyond the prostatic capsule (70.8%) and a significant number of patients had a node‐positive disease (18.1%). As the majority of patients had an extraprostatic extension and high malignant features (86.1% for Grade Group ≥3), there was an increased ratio of positive surgical margin status (41.9%). The median prostate volume of surgical specimens was 45 mL, with a median tumour volume percentage of 9.07%. The characteristics of the patients, which underwent histological examination of preperitoneal VDI, are summarized in Table 1.

**TABLE 1:** Clinical and pathological characteristics of patients with available preperitoneal vas deferens histology.

Characteristics	*N* = 332
Median age at RALP, years (IQR)	68 (63–73)
Median serum PSA concentration at diagnosis, ng/mL (IQR)	10.3 (7–18.7)
Median Briganti score, % (IQR)	19 (11.3–58)
Neoadjuvant ADT, *N* (%)	
Yes	25 (7.5)
No	307 (92.5)
Pathological tumour stage, *N* (%)	
pT2	97 (29.2)
pT3	234 (70.5)
pT4	1 (0.3)
Pathological lymph node status, *N* (%)	
pN0	272 (81.9)
pN+	60 (18.1)
ISUP grade at final pathology, *N* (%)	
2	46 (13.9)
3	129 (38.9)
4	19 (5.7)
5	138 (41.5)
Median prostate volume, mL (IQR)	45 (35–58)
Median tumour volume percentage in surgical specimen, % (IQR)	9.07 (4.07–17.14)
Surgical margin, *N* (%)	
Positive (R1)	139 (41.9)
Negative (R0)	185 (55.7)
Not assessable (Rx)	8 (2.4)
Distal VDI, *N* (%)	
Positive	20 (6)
Negative	312 (94)
Preperitoneal VDI, *N* (%)	
Positive	2 (0.6)
Negative	330 (99.4)

Abbreviations: ADT, androgen deprivation therapy; IQR, interquartile range; ISUP, International Society of Urological Pathology; PSA, prostate‐specific antigen; RALP, robotic‐assisted laparoscopic radical prostatectomy; VDI, vas deferens infiltration.

Regarding distal VD status, 20 patients (6%) had distal VDI, and the majority of them had simultaneous seminal vesicle infiltration (Table 2). Particularly for the pT3b stage, the finding of distal VDI was not associated with an increased possibility for nodal disease or positive margins (Table 3). Moreover, all 332 pairs of preperitoneal VD were examined for the presence of PCa infiltration, and positive findings were identified in two (0.6%) patients. The basic preoperative and postoperative characteristics of these cases are summarized in Table 4. Both patients had a highly aggressive disease, which was found in an already locally advanced stage. Distal VD was infiltrated bilaterally in both patients, while one patient had nodal disease.

**TABLE 2:** Distribution of VDI in cases with locally advanced prostate cancer.

Pathological stage	Number of cases	Distal VDI	Preperitoneal VDI
pT3a	104	1 (1% of pT3a)	1 (1% of pT3a)
pT3b	130	18 (13.8% of pT3b)	1 (0.7% of pT3b)
pT4	1	1 (100% of pT4)	0 (0% of pT4)

Abbreviation: VDI, vas deferens infiltration.

**TABLE 3:** Association of distal VDI positivity with nodal and surgical margin status in patients with pT3b pathology.

	Lymph node positivity			Surgical margin positivity		
Distal VDI	OR	95% CI	*P*	OR	95% CI	*P*
Positive (ref)	1	1	1	1	1	1
Negative	0.528	0.193–1.44	0.212	0.5	0.154–1.623	0.248

Abbreviations: CI, confidence interval; OR, odds ratio; VDI, vas deferens infiltration.

**TABLE 4:** Disease characteristics patients with preperitoneal VDI.

Patient	Case 1	Case 2
ISUP grade	5	5
Staging	pT3b, pN1, M0	pT3a, pN0, M0
PSA at diagnosis (ng/mL)	128	13
Briganti score (%)	58.7	9
Tumour volume (mL)	11.2	2.5
Tumour volume (%)	22	8.1
Clinical course	No recurrence	Bone metastases at 2 years
Laterality of preperitoneal VDI	Right	Left
Distal VDI	Yes	Yes
Postoperative therapeutic regiments	ADT Adjuvant RT	Adjuvant ADT and salvage RT Docetaxel and abiraterone
Running therapeutic regiment	ADT	Lu‐PSMA
Disease status	Stable	Progressing

Abbreviations: ADT, androgen deprivation therapy; ISUP, International Society of Urological Pathology; PSA, prostate‐specific antigen; PSMA, prostate‐specific membrane antigen; RT, radiation therapy; VDI, vas deferens infiltration.

The first case is a 68‐year‐old patient, who underwent a transrectal U/S‐guided biopsy for the investigation of an elevated PSA value (128 ng/mL). Histologic analysis showed adenocarcinoma of the prostate on both sides (left 100% and right 19%), Grade Group: 5. The patient had no major comorbidity, and staging through abdominal CT and bone scintigraphy was negative for distant metastases. After 3 months of neoadjuvant androgen deprivation therapy (ADT) with luteinizing hormone‐releasing hormone agonist (Leuprorelin 11.25 mg/3 months), he was treated with RALP combined with ePLND. The operation was performed transperitoneally, the lymph nodes of fossa obturatoria and external iliacal vessels were resected on both sides, while the preperitoneal parts of VD were sampled separately. The histological analysis reported a pT3b, pN1 (2/18), L1, V1, Pn1, R1 tumour Grade Group: 5 adenocarcinoma of the prostate, with additional infiltration of distal VD and seminal vesicles on both sides. Regarding the preperitoneal VD specimen, the sample of the right side was infiltrated, while both samples were not suspicious for PCa involvement radiologically in the preoperative setting and macroscopically during the RALP procedure. Because of the positive margin status, the patient underwent adjuvant radiation therapy for 3 months after the operation. During the 17‐month‐long follow‐up (FU) of the patient, there was no sign of clinical or biochemical recurrence.

The second case is a 56‐year‐old patient, who was diagnosed with PCa through transrectal U/S‐guided biopsy, after an elevation of PSA value to 13 ng/mL. Histology results reported the involvement of both sides (right 10% of the material and left 30%), 4/12 positive cores, Grade Group: 5 adenocarcinoma of the prostate, while the digital examination was normal. No signs of distant metastases were shown in the staging examination through abdominal CT and bone scintigraphy. The patient underwent a transperitoneal RALP with ePLND. The final histological report revealed a pT3a, pN0, pL0, pV0, Pn1, R1, Grade Group: 5 adenocarcinoma of the prostate. The additional analysis of preperitoneal VD showed an infiltration on the left side, a finding which was not detectable during preoperative imaging and intraoperatively. The patient was treated with adjuvant ADT (Leuprorelin 11.25 mg/3 months), and after the progression to castration‐resistant state and the exclusion of metastases through standard imaging (abdomen CT and bone scan), the patient was treated with salvage radiation therapy. Two years after the operation, the patient was restaged with PSMA–PET scan because of a PSA rise. The PSMA–PET scan demonstrated diffuse bone metastases, which prompted the initiation of chemotherapy (docetaxel) and second‐line hormonal therapy (abiraterone), while the ADT regimen was further administered. No increased radiotracer uptake was detected in the region of the excised preperitoneal VD. At the present (57‐month FU), the patient is treated with Lu‐PSMA, while the disease status is progressing.

## 4 DISCUSSION

In the present series of high‐risk PCa patients, a subgroup of 20 patients, mostly of pT3b stage, was found with infiltration of VD at a various extent. Distal VDI only was detected in 18 of the above patients, while two patients had additionally preperitoneal VDI. Among pT3b patients, distal VDI was not associated with a significant tendency for positive surgical margin or nodal disease. Both patients with preperitoneal VDI had prognostically adverse disease characteristics, but only one patient had PCa recurrence and progression.

The prevalence of distal VDI is low but not negligible, yet the histological analysis of distal VD is not recommended by the current guidelines for handling the radical prostatectomy specimen.^9^ Moreover, the extremely low prevalence of preperitoneal VDI in the above patient group shows that it is a rare manifestation of PCa local extension, which explains the fact that it is not investigated until now. In contrast to distal VD involvement, which is more often and represents a direct anatomical continuation of PCa local extension, preperitoneal VDI suggests a cancer cell transfer through VD lymphatics or lumen. Similar to the seminal vesicles, VD represents an accessory anatomical entity to the prostate gland, and it seems reasonable that PCa extension along VD may be characterized as further subclassifications of the pT3 stage, rather than subcategories of the pT4 stage because the latter relates to the infiltration of independent adjacent anatomic structures. A corroborating study for the extension over the lymphatics pathway was published at the beginning of the 19th century by two French anatomists, who supported the existence of lymph drainage of the prostate along VD to external iliac lymph nodes.^10^

This is the first report of histological proof of the above pattern of local extension in PCa, based on the systematic prospective sampling of preperitoneal VD after its division during RALP. There was only one report relating to the entire VD, which was based on novel imaging methods.^11^ In this report, the additional workup for the detection of the focus of biochemical recurrence after radical prostatectomy included imaging with PSMA PET/CT, which revealed an enhanced radiotracer uptake along the entire left intrapelvic VD, extending between the inguinal canal and prostatic bed as the main focus of the disease recurrence.

This unique type of local extension is possibly related to the developing testicular metastases during the course of PCa. Metastases from PCa to the testicles or proximal parts of the sperm transferring duct were already reported more than 40 years ago and mainly characterized patients with increased tumour burden.^12^ An autopsy study reported a prevalence of testicular metastases among men with PCa equal to 0.5%, while another study of histological analysis of the testicular tissue from patients, who underwent androgen deprivation orchidectomy for advanced PCa, resulted in a prevalence of 0.18% and presence of disease with atypical biologic behaviour and poor prognosis.^13^, ^14^ In the more recent series, testicular metastases are discovered through modern imaging methods, mainly PSMA PET/CT, in the context of detecting the focus underneath biochemical recurrence, and confirmed through histological analysis.^15^ A PSMA PET/CT‐detected testicular metastasis can be the solitary focus of disease recurrence, which may be diagnosed more than 10 years after the definitive PCa treatment.^16^

The hypothesis that PCa, mainly with high‐risk characteristics, may extend locally along VD up to the testicles may have important clinical implications for disease management. First, in high‐risk cases managed with radical prostatectomy, the routine transection of preperitoneal VD could contribute to the maximum oncological control of the disease. Additionally, for patients managed with radiation therapy as primary or salvage treatment, the intrapelvic VD may be included in the clinical target volume of radiation therapy planning. At present, the delineation of clinical target volume does not incorporate the preperitoneal VD.^17^ In order to find the independent prognostic value of preperitoneal VD resection, it would be required to sample the above specimen in larger patient series. However, it seems applicable to resect the preperitoneal VD during pelvic lymphadenectomy in high‐risk patients, because there is no additional morbidity from this operative step. Preperitoneal VD should additionally be considered as a possible focus of biochemical recurrence, and in this setting, it would be preferable to investigate the focus of biochemical recurrence through high‐accuracy methods, such as molecular imaging, because VD are very fine anatomical structures, which are inadequately assessed through conventional imaging. Lastly, preperitoneal VD resection may be indicated in patients with testicular metastases as a means to maximize tumour control, because preperitoneal VD represents the middle part of the path to the testicles and is expected to be infiltrated in such cases.

Regarding the limitations of our study, the small number of patients with preperitoneal VDI and the relatively short FU duration limited the feasibility of associating the above histological finding with the prognosis and the disease characteristics of PCa. This study highlights the existence of a new anatomical path of PCa local extension, which may introduce modifications in various settings of disease management because until now, there are no guidelines for the sampling and the importance of the histological status of VD in PCa patients.

## 5 CONCLUSION

PCa extension along VD may reach a more proximal point of VD than the reported from the existing data infiltration of VD adjacent to seminal vesicles. This rare manifestation of PCa local extension may be the intermediate step to the rare cases of recurrence in the testicles. However, more robust data are needed to confirm the aforementioned hypothesis. Distal VDI seems to have no additional prognostic value among patients with infiltrated seminal vesicles.

## AUTHOR CONTRIBUTIONS

*Conception and design*: Ali Serdar Gözen. *Data acquisition*: Antonios Koudonas and Samet Senel. *Data analysis and interpretation*: Samet Senel and Antonios Koudonas. *Drafting the manuscript*: Antonios Koudonas and Samet Senel. *Critical revision of the manuscript for scientific and factual content*: Ali Serdar Gözen, Jens Rassweiler, and Maurizio Colecchia. *Statistical analysis*: Samet Senel. *Supervision*: Ali Serdar Gözen and Jens Rassweiler.

## CONFLICT OF INTEREST STATEMENT

The authors report no conflicts of interest.

## Data Availability

The datasets generated during and/or analysed during the current study are available from the corresponding author on reasonable request.
